# Loneliness from the Perspective of Young People with Autism and/or ADHD: A Thematic Analysis of Adolescents’ Experiences and Understanding

**DOI:** 10.3390/children12101285

**Published:** 2025-09-24

**Authors:** Lily Verity, Suzanne Stewart, Stephen Houghton, Pamela Qualter, Leslie Macqueen, Simon Hunter

**Affiliations:** 1 Manchester Institute of Education, University of Manchester, Manchester M13 9PL, UK; lily.verity@manchester.ac.uk (L.V.); pamela.qualter@manchester.ac.uk (P.Q.); 2 The Graduate School of Education, The University of Western Australia, Perth, WA 6009, Australia; suzanne.stewart@uwa.edu.au (S.S.); leslie.macqueen@uwa.edu.au (L.M.); simon.hunter@gcu.ac.uk (S.H.); 3 Department of Psychology, Glasgow Caledonian University, Glasgow G4 0BA, UK

**Keywords:** ADHD, autism, adolescence, loneliness, thematic analysis

## Abstract

**Highlights:**

**What are the main findings?**

• Adolescents with autism and/or ADHD experience a complex relationship with loneliness and peer interactions and may not self-identify as lonely despite evident challenges in peer relationships.
• While the developed themes were similar to those of non-diagnosed youth, friendships provided significant functional support beyond emotional connection for autistic and ADHD youth.

**What is the implication of the main finding?**

• Autistic and ADHD adolescents’ nuanced relationship with loneliness highlights the need for greater sensitivity in how loneliness is conceptualized and assessed in neurodiverse youth.
• Strategies for intervention should prioritize the development of trusted friendships, peer inclusion, and social competence, as these are important for autistic and ADHD youth in navigating loneliness.

**Abstract:**

**Background/Objectives:** Loneliness is a common experience in adolescence, typically centered around difficulties in relationships with same-age peers and friends. It is often assumed that those diagnosed with autism and/or Attention-Deficit/Hyperactivity Disorder (ADHD) are at greater risk of loneliness than their non-diagnosed peers due to documented difficulties in making and maintaining friendships. Although quantitative research on loneliness and autism exists, there remains a notable gap in studies that explore the lived experiences of loneliness from the perspective of adolescents diagnosed with autism and ADHD, particularly in relation to their peers/or peers’ experiences. **Method:** To address this gap in the literature, 10 focus groups were conducted with adolescents diagnosed with autism and/or ADHD to discuss their experiences of loneliness. **Results:** Overall, young people with autism and ADHD did not consider loneliness to be a greater issue for them compared to their neurotypical peers. Six themes with five subthemes were developed through thematic analysis of the interview data: “not feeling like you belong when socializing”, “being alone can be a good thing but not when it’s not your choice”, “social media can be a good thing, but it’s not as good as in real life”, “not having anyone you can rely on to support you through tough times”, “school support can bring young people together”, and “sometimes it’s good to be distracted from negative thinking”. **Conclusions:** Although these themes are comparable to those emerging from research with non-autistic and non-ADHD youth, the importance of quality relationships with friends who provided support with daily functioning was viewed differently.

## 1. Introduction

Loneliness is characterized as an unpleasant emotional experience that arises when there is a discrepancy between an individual’s actual and desired social relationships [1]. It is described as involving sadness, anxiety, anger, and emptiness by those who have experienced it [2]. Across one’s lifespan, loneliness is associated with poorer mental and physical health [3], as well as with the onset of depression, anxiety, and self-harm [4], cardiovascular issues, and increased mortality [5,6,7,8]. The high prevalence of loneliness has led some scholars to declare a loneliness epidemic; consequently, loneliness has increasingly become a topic of interest for policy makers [9,10].

Evidence suggests that loneliness is more of an issue for youth than it is for other age groups [11], and that it is a particularly large concern for certain marginalized groups in society [12], including those with autism [13]. Autistic individuals experience higher rates of morbidity and mortality compared to non-autistic populations, potentially due in part to loneliness serving as a mediating factor [14]. Recent meta-analytic evidence also shows that young people with Attention-Deficit/Hyperactivity Disorder (ADHD) are also at increased risk of loneliness compared to their non-ADHD peers [15]. Despite this work, there is limited research that aims to understand the experience of loneliness among youth with autism and ADHD; therefore, in the current study, we set out to address this gap in the literature.

### 1.1. Developmental Trajectory of Loneliness

Loneliness is identified as following a U-shaped trajectory, peaking first in adolescence [7,16,17], a developmental period characterized by friendship instability [18]. Preferences for friendships are based on emotional support (i.e., someone to talk to) in adolescence compared to the need for physical companionship (i.e., someone to play with) in childhood. Developmental challenges such as identity formation also arise, where an individual ascertains their beliefs and values independently to those of their caregivers [19,20]. Therefore, it is not surprising that the social and emotional difficulties faced by adolescents explain why the prevalence of loneliness peaks during this period [21,22].

While loneliness in adolescence is a common experience, it is often transient [23]. It is theorized that when experienced transiently, loneliness can be adaptive, as it assures safety within a social group by motivating an individual to find where they fit in and are supported [24]. However, the developmental challenges of adolescents give rise to social sensitivity, which impedes upon their ability to reconnect when feeling lonely [25]. When loneliness is not overcome, it can become chronic and maladaptive, leading to detrimental behaviors such as hypervigilance and social withdrawal [3,26]. Persistent loneliness in young people is predictive of later depressive symptomology, particularly when it increases over time [27,28].

### 1.2. Autism, ADHD, and Loneliness

The developmental challenges of adolescents are experienced to a higher degree by those with autism and ADHD. These neurodevelopmental disorders share some symptoms such as difficulties in emotion regulation, attention, and social communication (see DSM 5, DSM 5 TR, 2013, 2022). According to Tannock [29], comorbidity/multimorbidity across and within NDDs is the rule rather than the exception. There is high comorbidity/multimorbidity between ADHD and autism (22–83% of autistic children and adolescents have comorbid ADHD, and 30–60% of children and adolescents with ADHD have symptoms of autism) [30]. A meta-analysis identified the prevalence of ADHD in autistic individuals as ranging from 50 to 70% [31].

Research shows that friendships are dictated by peers’ perceptions and that peers hold negative attributions about peers with ADHD [32]. Compared to NT peers, peer rejection is higher for autistic and ADHD adolescents [33]. Evidence suggests that young people with ADHD value fun and entertaining friendships, while their peers seek emotionally supportive friendships [34,35,36]. ADHD adolescents report having fewer friends than NT peers, and their friendships are seen to be less supportive and satisfying [37,38,39,40]. A recent meta-analysis (n = 15) found that ADHD youth report significantly higher levels of loneliness than non-ADHD youth [14]. Conversely, no differences were reported by Diamantopoulou et al. [41], Elmose and Lasgaard [42], Heiman [35], and Houghton et al. [43]. While ADHD youth have fewer friendships compared to their peers, they also have lower expectations of friendships (i.e., valuing mutual entertainment rather than emotional support) and thus do not experience greater levels of loneliness [44].

For autistic young people, the findings have been more consistent with higher levels of loneliness reported [45,46,47,48]. A meta-analysis [49] revealed increased loneliness in the autistic population from childhood to adulthood compared to neurotypical individuals. Autistic adolescents report a desire for friendships and value friendships that are centered around companionship, i.e., spending time together [50,51,52,53]. Autistic young people are known to have difficulties making and keeping friends [54], and their understanding of friendship differs from that of their neurotypical (NT) peers [55]. Autistic youth face difficulties understanding social rules [56] and perceive friendship differently to their NT peers, which makes it more difficult to fit in [57]. Autistic youth report lower acceptance, companionship, and reciprocity in the social networks they are involved in compared to their NT peers [55]. The poorer quality friendship autistic adolescents experience in comparison to their neurotypical peers [58] is often attributed to a preference for alone time and difficulty making and maintaining friendships [59]. However, it should be noted that being alone is not the same as loneliness, and a positive view of alone time makes loneliness less likely [60], and lack of access to friendships for autistic adolescents is shown to result in loneliness and decreases in wellbeing for autistic youth [51,52,61,62].

There is a lack of research that aims to develop an understanding of loneliness amongst autistic and ADHD youth. To enhance our understanding of the link between autism, ADHD, and adolescent loneliness, qualitative research is essential. While quantitative studies highlight associations with loneliness, they fail to provide insights into the social worlds of autistic and/or ADHD youth that can help explain these associations. This understanding is crucial for developing effective interventions that alleviate loneliness in this group. Previous qualitative research has shown that autistic children and adolescents understand the concept of loneliness [63,64]. However, there is a significant gap in the research exploring whether autistic/ADHD adolescents identify themselves as experiencing loneliness and how they perceive their experiences compared to their NT peers.

Qualitative research in Japan has identified loneliness as a prevalent theme in friendships of young people with autism. Autistic youth who experienced loneliness reported feeling disconnected in group settings, particularly when their close friends were not present [46]. However, research is needed that asks autistic youth directly about their loneliness experiences. To address this, the current study explored autistic and ADHD adolescent’s experiences of loneliness through a thematic analysis of qualitative semi-structured interviews. By delving into their personal narratives, we aimed to uncover the nuanced social dynamics and contexts that contribute to feelings of loneliness, thereby providing valuable insights for interventions.

## 2. Materials and Methods

The use and success of focus groups for exploring a wide range of sensitive and non-sensitive issues with neurotypical children and adolescents is well documented in the contemporary research literature [65,66,67,68,69,70,71,72]. Relatively few focus group studies have been conducted with neurodivergent youth. However, those that have been undertaken have generated important information on a range of topics, including sexuality and relationships among adolescents [73,74], the challenges of high school [75], managing medication [76,77,78], emotion dysregulation [79], technology support [80], and quality of life [81]. To date, however, the subjective interpretations of feelings of loneliness among neurodivergent adolescents appear to not have been investigated.

For the current study, 10 focus groups were conducted with 36 young people, aged 10 to 16 years, who had been diagnosed formally by a pediatrician or child psychiatrist with autism or ADHD. The participants were from mainstream primary and secondary schools in Perth Metropolitan, which covers a diverse range of socio-economic statuses. Of these participants, 22 were male and 11 were female. Further details regarding the demographics of each group can be found in Appendix A.

### 2.1. Procedure

The focus groups used a standard format with semi-structured questions to explore the views of autistic and ADHD adolescents about loneliness and worry, although the current study deals only with where focus groups discussed loneliness. Each of the focus groups were presented with an ‘alien’ scenario to capture participant’s attention and contextualize the focus of the initial questions: “The focus of our group today is going to be ‘loneliness’. Imagine that a group of aliens has just landed on Earth. These aliens know very about life on Earth. They don’t know how a person your age feels and thinks.” The remaining focus questions were then asked (see Appendix B).

Prior to the research, ethics approval had been obtained from the Human Research Ethics Committees of the University of Western Australia (#2019/RA/4/20/6130), the Western Australian Department of Education (#D18/0383437 and #D23/1249905), and the principals and/or deputy principals of the participating schools. After indicating their interest, schools nominated a contact person to liaise with the researchers. Each school was sent an overview of the research, information sheets, and consent forms. These were subsequently sent to the parents of children with autism and/or ADHD identified by the schools, inviting them to allow their child(ren) to participate in the focus groups.

The times selected for the focus groups were decided by the respective schools, such that they had limited impact on normal day-to-day school activities The focus groups were subsequently held in the schools in environments familiar to the participants (e.g., a comfortable room on the school campus such as the school library or a room with beanbags). School staff who were familiar to the participants were present for either part or all of the focus group. The groups consisted of neurodivergent peers in order to establish comfortability. The participants were engaged and open during the focus group sessions. The focus group interviews were carried out by three members of the research team representing varying levels of academic experience: a research assistant, a doctoral researcher, and a senior academic.

The researchers were with the participants in the designated room at the pre-arranged times and they initially engaged them with informal chat. The participants were made aware of a microphone strategically placed to record their responses and were asked if this was acceptable. The participants were informed that if anyone objected to the microphone, no recordings would be made. At the outset of each interview, the participants were reminded that there were no right or wrong answers to the questions and that they could withdraw at any time without having to give a reason. None chose to do so. Reassurances were given regarding the confidentiality of any information shared and that only the researchers would have access to the interviews to check on the accuracy of reporting and questioning style. Following a few minutes of general conversation, the researcher commenced the focus group using a conversational style to encourage and maintain the flow of discussion and to ensure that the responses were instinctive and unbiased.

The participants were encouraged to express their opinions, and the researcher continued to probe their responses with follow-up questions where appropriate [82] until it was determined that no new information was being offered. Then, the next question was asked. If the participants digressed from the question, they were allowed to continue for a time before being gently guided back to the original point. On average, each focus group lasted for approximately 40 min (ranging from 30 min to 55 min). To ensure that all students were treated equally, all information was reported accurately, and the researcher adhered to all codes of behaviors and conduct during the focus groups, 30% of the recorded focus group interviews were reviewed by a second researcher who had been involved in conducting the focus groups. The researcher had 20 years of experience in conducting focus groups with young people and those with autism and ADHD specifically. No concerns or issues regarding the focus groups were raised by the second researcher, who attested to the accuracy and conduct of the interviewer/interviews.

### 2.2. Data Analysis

A framework approach to thematic analysis was conducted to evaluate the transcripts. The aim was to develop themes that described the salient issues discussed by participants across the dataset. This involved each member from the analysis team reading through and summarizing each transcript to become familiar with the data; those involved in conducting the focus groups added their impressions based on the additional context they had from that experience. Following this, open coding was conducted, and an initial coding framework was developed by LV, LM, and SS and shared with the rest of the team for edits and feedback. Initial codes were trialed on two transcripts and edited to better fit the dataset. Based on feedback from the trial, edits to the framework were made to address where disagreements between coders occurred. The edited framework was then applied to the whole dataset within NVivo. Using Excel, coded data were mapped into a framework matrix to help aid interpretations by presenting data in a visually accessible way. An initial list of themes was developed by LV and shared with SS who provided detailed feedback incorporating their expertise from working with adolescents diagnosed with autism and ADHD. From this, the finalized themes were created, which consisted of 6 main themes with 5 subthemes.

## 3. Results

Ten focus groups were held with autistic and/or ADHD youth to discuss the experience of loneliness in their age group (10–16 years). Overall, they did not see loneliness as a problem that was more pertinent to them than their NT peers. In general, they considered loneliness as more impactful on young people—teenagers in particular—than on adults. Six overarching themes and five subthemes were developed from the focus group participants’ discussions of loneliness.

**Theme** **1.** Not feeling like you belong when socializing.

**Theme** **1a.** *Having different interests from the people you spend time with.*

The participants talked about loneliness occurring in social situations where they did not feel comfortable with those around them. Finding friends was particularly difficult if the people they were with had different interests to them and wanted to spend their time differently.

“You’re in a room with people but they have completely different interests, and you just feel lonely inside because no one will be your friend and share the same interests.” (13–14 years, autism/ADHD)

**Theme** **1b.** *Having that person whom you trust and understand.*

The participants considered good friends to be those who understood them, were trustworthy, and supported them. The importance of that friend they could rely on was evident when they discussed days when that friend was absent.

“If my best friend was away, I would have no one to talk to. I have another friend, but they might not understand [me] like my best friend does.” (13–14 years, autism/ADHD)

“My two friends, who I usually go to the lockers with and talk to they were on [excursion], so I felt a bit lonely walking too…” (14 years, autism)

**Theme** **1c.** *Being excluded by classmates.*

In some cases, the participants talked about how they felt about their peers treating people with disabilities differently, making them feel excluded.

“A lot of people get excluded because they have something wrong with them, like a disability or they look different to other people, then they get excluded from those people.” (13–14 years, autism/ADHD)

“[…] They feel like they’re different from everyone else because of their disability or how they look; they look different, so they won’t fit in […]. It makes it harder for them to feel like they can talk to someone.” (13–14 years, autism/ADHD)

**Theme** **2.** Being alone can be a good thing, but not when it’s not your choice.

**Theme** **2a.** *Being comfortable in alone time, but not loneliness.*

The participants talked about how, sometimes, they would be happy to be alone, taking time to do the activities they wanted to do by themselves. They thought enjoying time alone could help them self-reflect; ultimately, once they returned to spending time with others, they would feel better equipped to socialize.

“Sometimes, you just want to sit back and just be alone to think about like your thoughts.” (14 years, autism)

“You can’t enjoy other people’s company unless you enjoy your own company.” (12–16 years, autism/ADHD)

The participants reported that their comfortability with being alone could mean they felt less lonely than their peers:

“Others can feel really bad when they’re lonely. I tend to not really care if I’m lonely because there are so many strategies of being alone.” (10 years, autism)

Others acknowledged that whilst they might experience loneliness less, it may be felt more intensely by them compared to their neurotypical peers:

“It’s sort of like my ADHD and autism cause, I mean, it boosts my sense, I think […]. It sort of makes me, like, feel more lonely when I get lonely.” (10 years, autism)

**Theme** **2b.** *Wanting to be with others but having no one to spend time with.*

The participants talked about how loneliness arose when they had no one to talk to, play with, or spend time with when they wanted to socialize. They talked about times during the school day when loneliness was commonplace (break times or at times when they were separated from friends). During these times, it was expected that everyone would be with friends, and it would be difficult for those who had no one to talk to. Outside of the school day, they talked about feeling lonely before and after school when they went from having lots of friends around them to being alone at home.

“Generally, I feel lonely at recess and stuff if no one is playing with me. But that’s only sometimes, because I still play with people sometimes.” (11 years, autism)

“I feel lonely before and after school because I’m an only child and I know I have, like, nothing to do.” (10 years, autism)

The participants also discussed the difficulty of balancing the desire to be with others with their desire to spend time alone:

“Part of my brain just tells me, like, I don’t want to, I’m like it’s sort of hard to find people to play with, I guess” (10 years, autism)

**Theme** **3.** Social media can be good for chatting with friends, but it’s not as good as in real life.

Early-aged adolescents spoke about gaming as a way of making connections, communicating, and having friends, whereas the older participants focused on social media. It was evident that socializing online was not viewed as good as spending time with friends in-person.

“If you’re on social media, you feel like you shouldn’t be using social media. You should be spending time in person. So, being on your phone and apps all day, it’s not socializing very well.” (12–16, autism/ADHD)

The participants noted that they missed out on facial expressions and could sometimes misinterpret neutral messages or lack of replies, such as their friends being angry with them.

“I have different friends online, so, sometimes, I’ll text them and wait for them to reply. They won’t reply and then I’ll start worrying, like thinking they don’t care about me […].” (13–14 years, autism/ADHD)

Social media could also contribute to problems continuing for young people, particularly older adolescents, at school where they felt left out or ostracized.

“I think, if you see posts with people and they’re hanging out with other people and you’re not, it’s like your friends hang out somewhere else.” (14 years, autism)

“The most I can think of is if someone starts trash-talking you publicly to other people and they send out an image saying like, ‘oh, this person’s ugly and stupid and they’re different from me, so I hate them for that’.” (13–14 years, autism/ADHD)

**Theme** **4.** Not having anyone that you can rely on to support you through tough times.

Young people talked about how loneliness involved having no one who they could open up to, express themselves with, or who could provide them with comfort and reassurance. Friends seemed to provide a particularly important role at this age, and young people thought it was important to have friends who cared about them.

“I would describe loneliness as not having friends [or] family who care about you […], no one who can give you their kindness, love.” (13–14 years, autism/ADHD)

“[...] Because if you lose all your family members and you don’t have anyone to turn to, the best people you can turn to are your friends–if you have no one else, you will basically be miserable, sad, and lonely.” (13–14 years, autism/ADHD)

**Theme** **5.** School support can bring young people together.

The participants reported ways their schools had helped them feel cared for, such as bringing people together in gaming clubs and pastoral care groups.

“[...] Put on more activities and more group activities […] to bring people together […] and people who don’t really hang out with each other—maybe they can become friends.” (12–13 years, autism)

They talked about how those within the school, such as teachers and peers, could help those feeling lonely by providing them with support and making efforts to include them. Having teachers that cared about their welfare and who they “clicked” with could help them talk more about their feelings.

“I had someone come up to me and say ‘are you lonely, do you want some friends?’ I said ‘yes, please’.”(13–14 years, autism/ADHD)

[A good teacher] “gives advice when you don’t ask for it […]. The teacher asks if you’re okay.” (12–16 years, autism/ADHD)

**Theme** **6.** Sometimes it’s good to be distracted from negative thinking.

The participants talked about the ways they would distract themselves from the negative thoughts and feelings that arose when they felt lonely. These strategies were not used to ignore their feelings of loneliness but to get through them. Distractions helped young people take their minds off things. For example, one participant talked about how listening to music or drawing “doesn’t help me being lonely, but it helps me feel better.”

“It [gaming] takes your mind off everything. You don’t feel lonely […], it takes you away from reality.” (12–13 years, autism)

## 4. Discussion

In our introduction, we highlighted the inconsistent findings that have emerged from empirical research regarding loneliness among adolescents, including those with neurodevelopmental disorders such as ADHD and autism. While quantitative studies have provided important information, they have not offered much on adolescents’ subjective insights into loneliness. This present study sought to address this via a series of interviews, the findings from which revealed six overarching themes and five subthemes, each providing critical insights into the experiences of loneliness among adolescents with autism and ADHD.

These insights are important because adolescents with autism and ADHD often experience great difficulty navigating the daily social and emotional landscapes in their lives and, as a result, often withdraw and become lonely. This is hardly surprising, given that the core behaviors and symptoms of ADHD (e.g., hyperactivity/impulsivity, struggling to focus) and autism (e.g., social communication difficulties affect the ability of adolescents with ADHD and/or autism to understand and respond to social interactions, a critical skill for adjusting their own behavior [83,84]. This often leads to social interactions that are characterized by disruptive, intrusive, and aggressive behavior [85,86], which, in turn, results in feelings of disconnection, peer rejection, social isolation, and loneliness for adolescents with ADHD and autism [87].

However, contrary to the prevailing assumption that adolescents with autism and ADHD are at heightened risk of loneliness, those in this present study did not perceive themselves as experiencing loneliness any more frequently than their NT peers, although some acknowledged that when they did feel lonely, it may be felt more intensely. Although adolescents with ADHD have great difficulty establishing and maintaining friendships in early adolescence, by mid- and late adolescence, this lessens [88] and, as a result, feelings of loneliness also decline. With reference to adolescents with autism, our findings are at odds with the empirical evidence, including a meta-analysis by Hymas et al. [49] and a systematic review of loneliness in young people with autism [89]. Hymas et al. [49] identified 23 studies involving 10- to 65-year-olds; they reported that all 23 studies found increased loneliness in autistic compared to neurotypical samples (significant difference in 21 studies). Furthermore, Papagavriel and Bathelt [89] found that, in 15 studies, children and adolescents with autism were lonelier than their non-autistic peers. It is known that individuals with autism can find it difficult to describe internal emotional experiences (alexithymia) and that they experience issues related to self-awareness [90], resulting in under-reporting of loneliness; this may explain why, on the whole, the participants in our study did not perceive themselves to be any lonelier than their NT peers. It is also possible that discussing loneliness in a group setting influenced how openly participants reported their experiences. At the same time, this study represents one of the few qualitative investigations of loneliness among autistic and ADHD youth, and it may be that quantitative measures do not fully capture the ways in which loneliness is experienced by neurodivergent adolescents.

While not perceiving themselves as lonelier than their peers, these adolescents often felt excluded by their peers. Up to 50% of children and adolescents with ADHD experience peer rejection from their classmates [91], primarily because they are judged as “annoying”, “boisterous”, “irritating”, and “intrusive” [92]. Furthermore, difficulties with cooperative play, responding to social cues, and self-regulation add to the risk of social isolation [93]. Adolescents with autism are like their adult peers, who also describe being on the “outside” of social experiences. This may be because of a need for alone time due to the emotional and cognitive strain from everyday activities, rather than loneliness [94].

Having stated this, young people have their own unique social restructuring and life events that impact loneliness differently from adults [95]. For adolescents with autism, feelings of being on the outside may be because they have a lower desire or motivation to interact with peers [13] or to make friends compared to their non-autistic peers. Nevertheless, the adolescents in the present study qualified this by adding that their preference for spending time alone was context-dependent, and although solitude was welcomed when self-chosen, it could exacerbate feelings of loneliness when imposed by circumstances. This duality underscores the complex interplay between the desire for social connection and the need for individual space, reflecting a broader tension in the social lives of autistic and ADHD youth.

For some autistic and ADHD adolescents, insufficient connections to others can lead to profound and long-standing negative consequences, while having quality friendships can provide them with numerous social and emotional benefits [96,97,98]. Adolescents with ADHD have lower quality friendships than those without ADHD [98,99,100,101], while the lower quality of friendships in autistic adolescents is also well established [45,48,58,,102]. The participants in the present study reported having one close friend. They also highlighted that loneliness was most acutely felt during the absence of this friend, particularly during school breaks or other structured social contexts. This underlines the important role that close friendships play for ADHD or autistic youth; in the current study, they relied on friends to provide stability in their school life by accompanying them in their daily routines.

The quality—rather than the quantity—of friendships is critical to psychosocial functioning [103], but adolescents with ADHD rely on their best friends for amusement, fun, and mutual entertainment, whereas adolescents without ADHD view their best friends for emotional support, caregiving, and intimacy [35]. This mismatch in salient features of quality friendships can make maintaining friendships difficult for ADHD youth, which is significant, because quality friendships are protective against psychological symptoms and mental health difficulties [104,105,106]. Similarly, autistic children and adolescents have poorer friendship quality due to their different social preferences and communication style [102]. Those social preferences were reflected in the current study, in which even when participants acknowledged their need for time with friends, they felt challenged by the energy it took to socialize. In adolescence, neurotypical youth report a preference for high frequency of social context, which may be at odds with autistic and ADHD youths’ need for alone time as respite [107].

Whilst research has typically focused on improving social skills in autistic and ADHD youth, this may heighten stigma and encourage masking of differences without necessarily leading to social acceptance [108]. An alternative approach is to acknowledge the dominancy of stigma against neurodiversity in society [109]. Research with stakeholders identifies the need for interventions to enhance awareness, knowledge, and understanding of neurodiversity in educational settings [109]. In addition, youth in the current study identified ways the school could address loneliness, such as by organizing group activities and through pastoral support from staff.

The early-aged adolescents in this study discussed video gaming in the context of making connections, having friends, and communicating, whereas the older adolescents focused on social media. It is posited that video games allow young people with autism and ADHD to escape from the frustrations and failures of real life, experience success and achievement in a virtual world [110], facilitate social connection, build new friendships, and sustain existing ones [111,112] Furthermore, according to Cummings et al. [113] social media/internet use displaces other types of communication and leads to a negative effect on friendship quality (i.e., the displacement hypothesis). Conversely, Lee [114] argued that social media supplements offline communication and increases overall time spent in contact with others (i.e., the increase hypothesis). Research by van Schalkwyk et al. [115] provided support for social media use being associated with better friendship quality in autistic youth (i.e., the increase hypothesis). The adolescents in the present study thought that socializing online was not as good as spending time with friends in-person and that they could misinterpret neutral messages or delays/lack of replies in a negative manner. In previous research, it has been postulated that social media may be better suited to the unique communicative styles of those with NDDs, with less reliance on non-verbal information such as interpreting facial expressions [116]. Whilst social media may allow for non-verbal communication that may be preferable in certain contexts, the current study highlights that social media still provides social challenges for neurodivergent youth.

### 4.1. Implications and Future Directions

The findings of this study contribute to a deeper understanding of the mechanisms underlying loneliness in autistic and ADHD adolescents and have implications for both practice and research. The protective role of trusted friendships and the contextual relevance of solitude mirror patterns observed in NT adolescents, suggesting areas of commonality in social experiences. However, the unique challenges posed by online social interactions reveal a specific area where autistic and ADHD youth may benefit from tailored interventions. Future counseling and educational programs could incorporate strategies to help adolescents interpret and respond to digital communications, thereby enhancing their confidence and reducing social anxiety in online environments. It is critical that further research is conducted into social media use by autistic and ADHD youth, as they are a vulnerable population at risk of cyberbullying, exposure to inappropriate content, and less meaningful interaction than face-to-face engagement.

This is particularly important, given the intersection of online and offline social experiences and the increasing prevalence of digital communication among adolescents. Additionally, interventions aimed at fostering peer inclusion, developing social competence, and supporting the cultivation of trusted friendships are essential for promoting social wellbeing. By addressing those areas, practitioners and researchers can contribute to reducing the risk of persistent loneliness and improving quality of life for autistic and ADHD youth.

### 4.2. Limitations of the Research

Although this qualitative study recruited a large sample (n = 36) and conducted many focus groups (10), several limitations must be acknowledged. Although all participants in the present study had a formal neurodevelopmental disorder (NDD) diagnosis, none of them had a diagnosed intellectual disability. The unique challenges that adolescents with ADHD or autism and an intellectual disability would face in friendship development might be different; therefore, the present findings are specific to adolescents with ADHD or autism without an intellectual disability.

The research focused on Western Australian adolescents diagnosed with autism or ADHD, so the findings cannot be generalized to the wider population of other NDDs, such as intellectual impairment, motor disorders, or communication disorders. Future research should, therefore, recruit a more diverse population of adolescents with NDDs, e.g., those with communication disorders, motor disorders, and intellectual disabilities.

The current study presents findings from focus groups with adolescents diagnosed with autism, ADHD, or both. Owing to the nature of focus groups and the analytic approach used, it was not possible to attribute specific findings to participants with a singular diagnosis versus those with a dual diagnosis. Although autism and ADHD are highly comorbid, it is important to acknowledge that the findings may not be equally applicable to all autistic and ADHD adolescents.

Additionally, the time that had elapsed between data collection and analysis made it unfeasible to conduct member checking, leaving it uncertain whether participants would endorse the research team’s interpretations. Nonetheless, efforts were made to ensure qualitative rigor by thoughtfully representing the voices of young people through the inclusion of numerous quotes drawn from across the focus groups.

## 5. Conclusions

In conclusion, this present study underscores the importance of understanding loneliness through the lived experiences of autistic and ADHD youth. Listening to autistic and ADHD adolescents has offered valuable insights into loneliness in a highly vulnerable population. This has enhanced our understanding, which is particularly valuable for designing interventions to destigmatize neurodivergence and develop neurodiversity-affirming policies such as neurodivergent-inclusive social programs and autism-aware education that align with the unique social and emotional needs of this population and ultimately enhance their social integration and mental wellbeing.

## Data Availability

The data presented in this study are available on request from the corresponding author. The data are not publicly available due to confidentiality.

## Appendix A

### Appendix A.1. Focus Group Characteristics

Group 1	autism	14 years
Group 2	autism/ADHD	13–14 years
Group 3	autism	10 years
Group 4	autism	14 years
Group 5	autism/ADHD	15 years
Group 6	autism	12–13 years
Group 7	autism/ADHD	12–16 years
Group 8	ADHD	12–13 years
Group 9	autism/ADHD	13–14 years
Group 10	autism	11–12 years
Note: Participant numbers and gender per group have been omitted to avoid statistical disclosure.		

## Appendix B

### Appendix B.1. Focus Group Protocol

The protocol shows questions asked during the focus groups.

	Focus Group Introduction and Questions Read out: “The focus of our interview today is going to be ‘worry * and ‘loneliness’. Imagine a group of aliens just landed on Earth. These aliens don’t know very much about life on Earth. They don’t know how a person your age feels and thinks.”
	Q 1: How would you explain to them what worry is? (This is then repeated but loneliness is substituted for worry.)
	Q 2: How do you think someone your age who is worried/lonely feels?
	Q 3: What does someone your age who is worried/lonely think about their situation? Q 4: Are there any differences between boys and girls who feel worried/lonely?
	Q 5: Have you noticed any differences between people your age who feel worried/lonely and adults who feel worried/lonely? Q 6: Do you worry or feel lonelier than others your age? Q 7: Are there any differences in feeling lonely between people with and without ADHD/ ASD/SLD (depending on group being interviewed)? Q 8: Does having ADHD/ASD/SLD make you feel more lonely? Q 9: Are there any times or any places before school, during school and after school where adolescents worry about things/feel lonely?
• Why do you think adolescents worry/feel lonely in this situation? • How do you think these adolescents might feel at the time? • What is it that’s happening in the situation (at the time) that makes them worry/feel lonely?	
	Q 10: I was talking to a group of adolescents your age and they mentioned they worry when doing other things, such as using social media and being online. What do you think about that?
	Q 11: Are there any other times you can think of when adolescents worry or feel lonely?
	Q 12: Do you think someone can be alone and not feel lonely? Can you think of any situations?
• What about the other way around? Can someone not be alone and feel lonely? Why? Can you think of any situations?	
	Q 13: What advice would you give to someone in your class who feels lonely?
• Could you help them? How might you do that? Or, why not? What might make it difficult for you to do anything to help?	
• Can teachers and schools help them? How might they do that? Or, why not? What might make it difficult for them to do anything to help? • Could you help yourself if you felt lonely? How might you do that? Or, why not? What might make it difficult for you to do anything to help? • Who might you turn to?	
* The focus groups discussed both worry and loneliness, the current study pertains only to discussions of loneliness. Only data that discussed loneliness was coded and included in the current study.	
